# Supplementary figures and images for: Causality of anthropometric markers associated with polycystic ovarian syndrome: Findings of a Mendelian randomization study
Source: PLoS One. 2022 Jun 9;17(6):e0269191. doi: 10.1371/journal.pone.0269191 (PMC9182303; doi:10.1371/journal.pone.0269191)

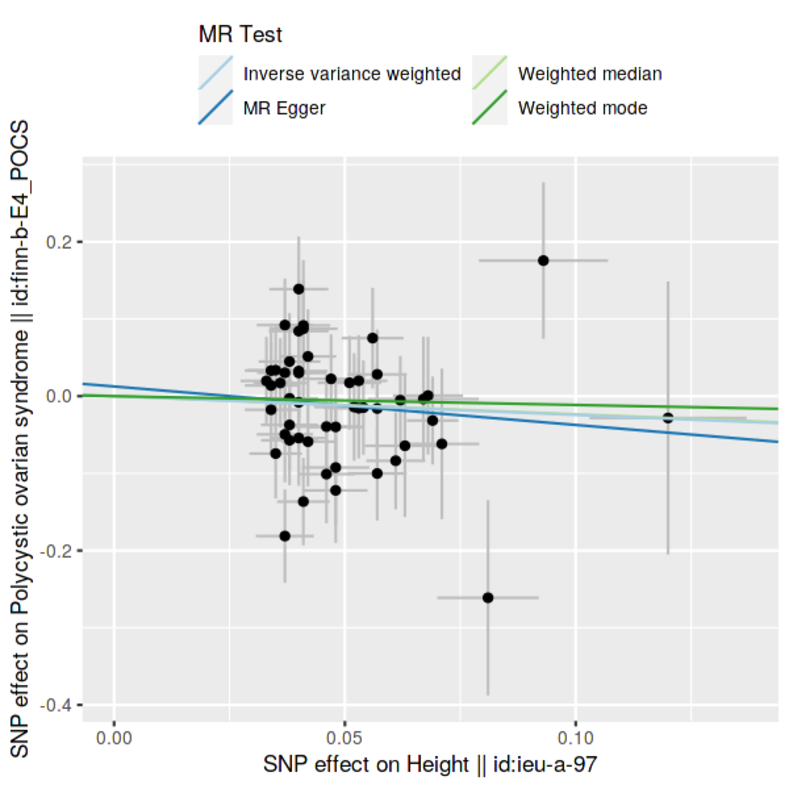

Supplement: S1 Fig — Trend lines from the four different two-sample Mendelian randomization methods employed, are also included in each scatter plot. (TIF) [file pone.0269191.s001.tif]

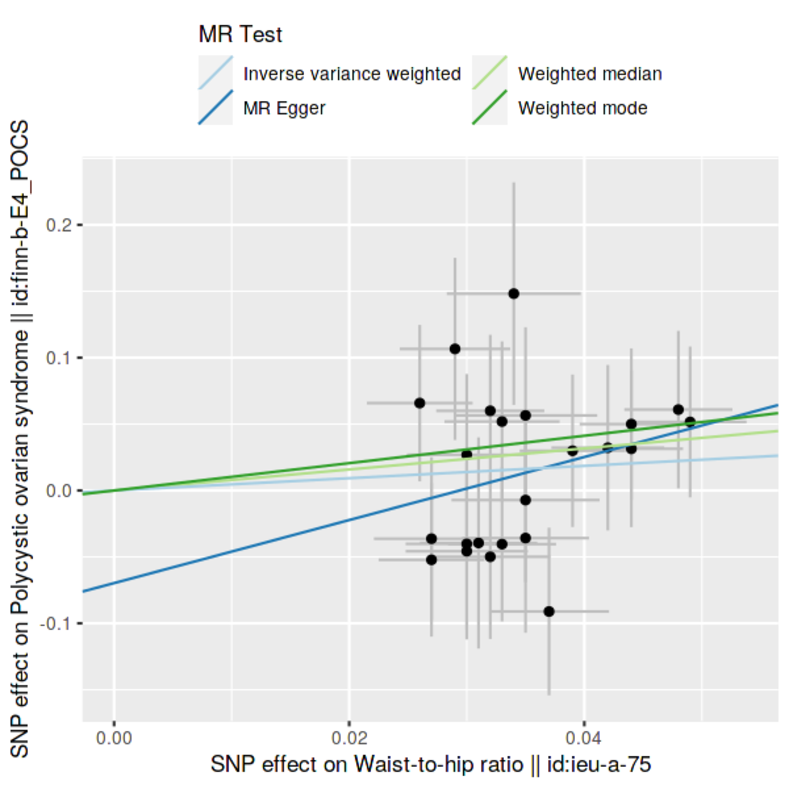

Supplement: S2 Fig — Trend lines from the four different two-sample Mendelian randomization methods employed, are also included in each scatter plot. (TIF) [file pone.0269191.s002.tif]

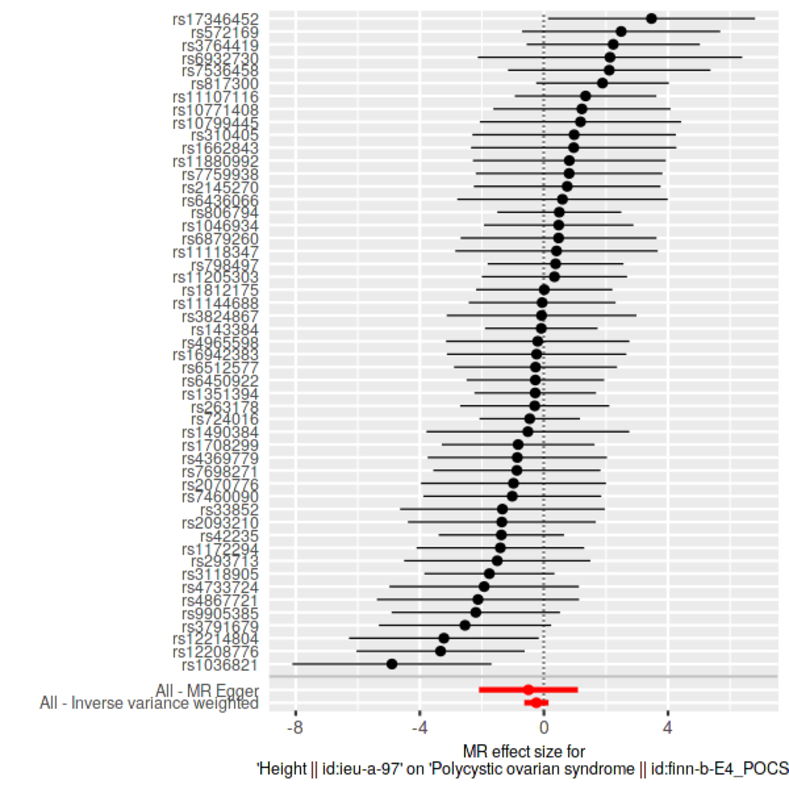

Supplement: S3 Fig — Effects of individual SNPs and pooled estimates from MR-Egger- and inverse variance weighted methods are visualized. (TIF) [file pone.0269191.s003.tif]

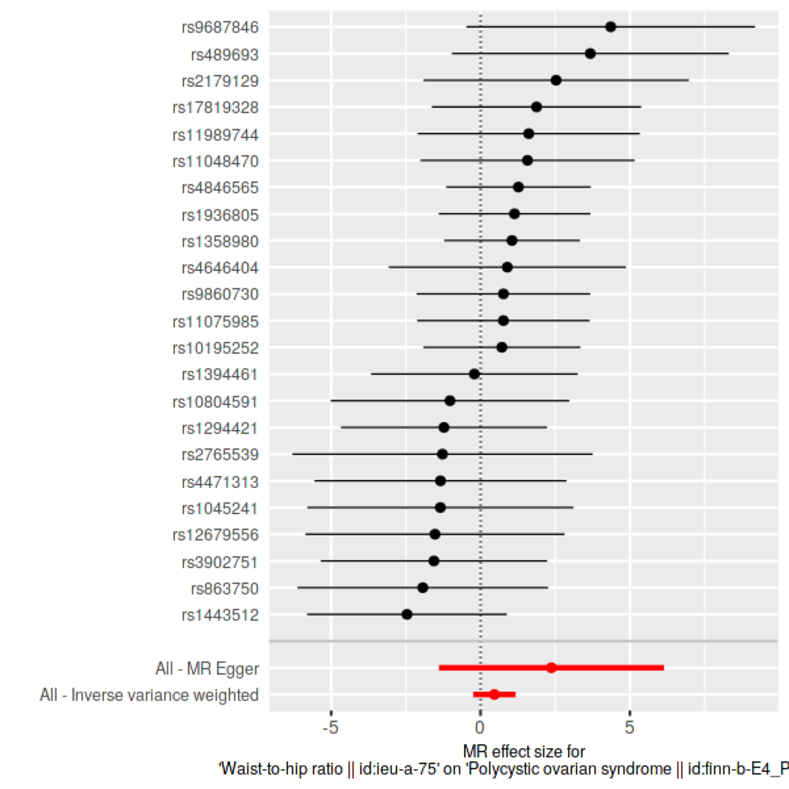

Supplement: S4 Fig — Effects of individual SNPs and pooled estimates from MR-Egger- and inverse variance weighted methods are visualized. (TIF) [file pone.0269191.s004.tif]

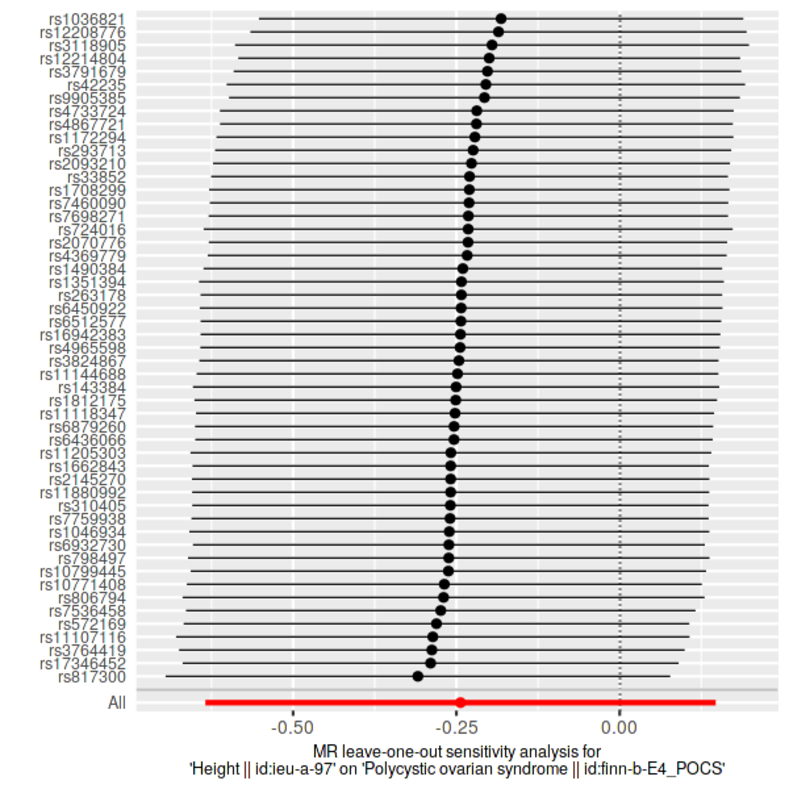

Supplement: S5 Fig — A given dark point indicates the effect measure from inverse variance weighted Mendelian randomization analysis excluding that specific SNP. The red lines indicate pooled analyses encompassing all SNPs. (TIF) [file pone.0269191.s005.tif]

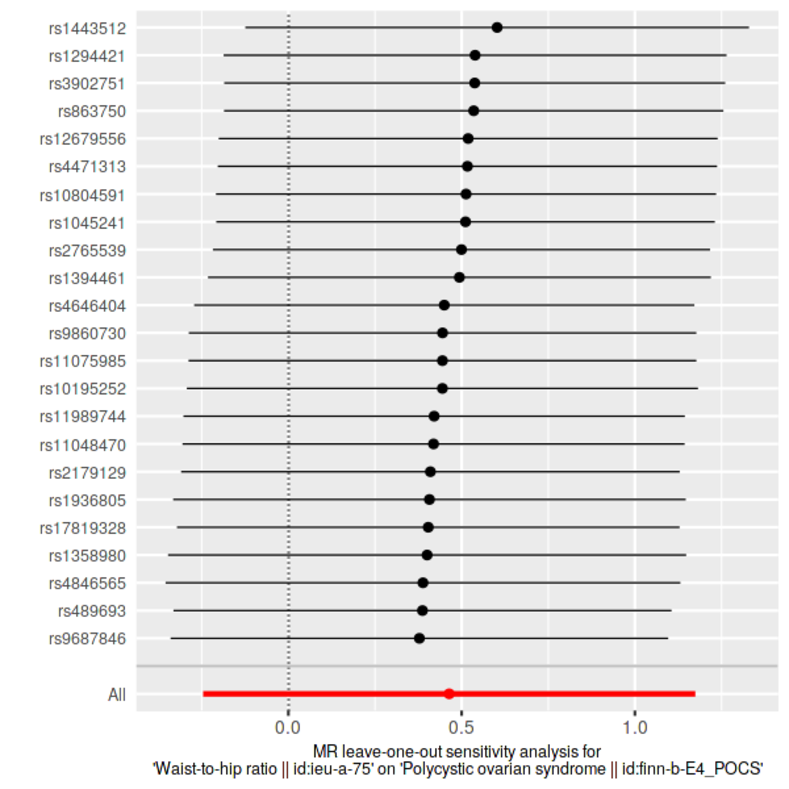

Supplement: S6 Fig — A given dark point indicates the effect measure from inverse variance weighted Mendelian randomization analysis excluding that specific SNP. The red lines indicate pooled analyses encompassing all SNPs. (TIF) [file pone.0269191.s006.tif]

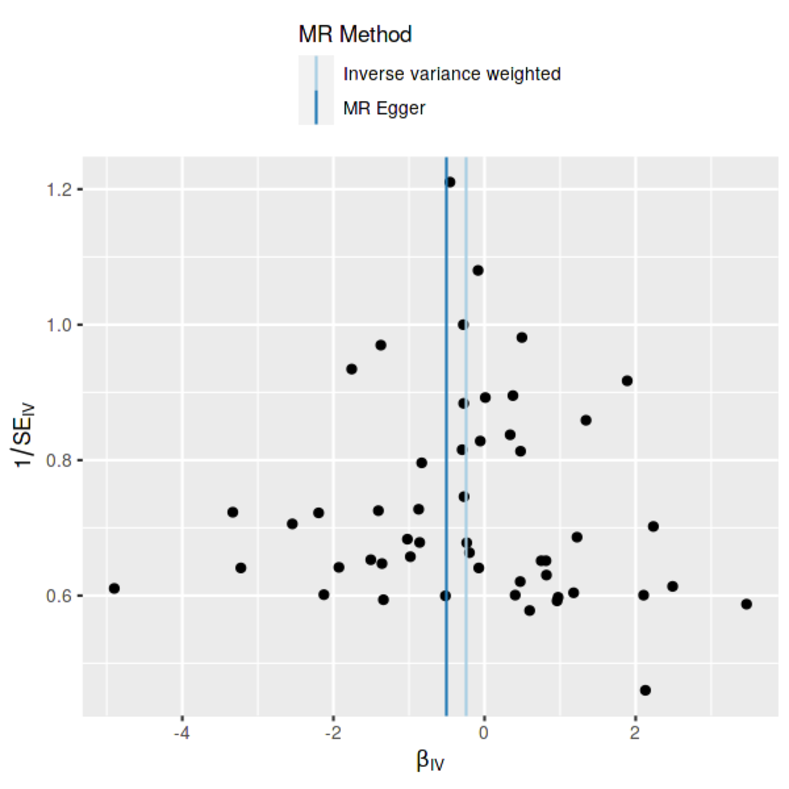

Supplement: S7 Fig — (TIF) [file pone.0269191.s007.tif]

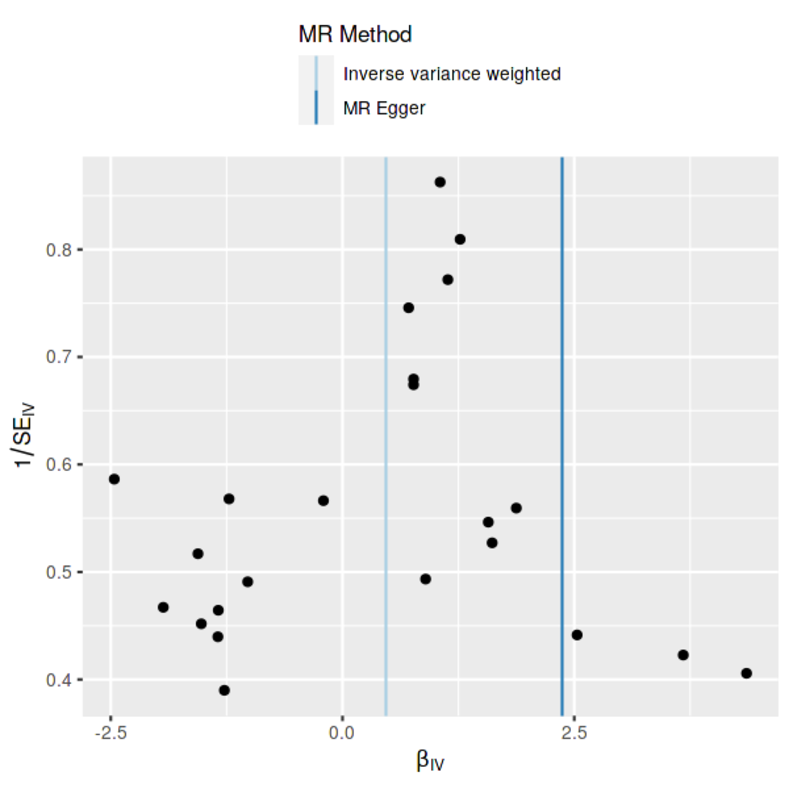

Supplement: S8 Fig — (TIF) [file pone.0269191.s008.tif]

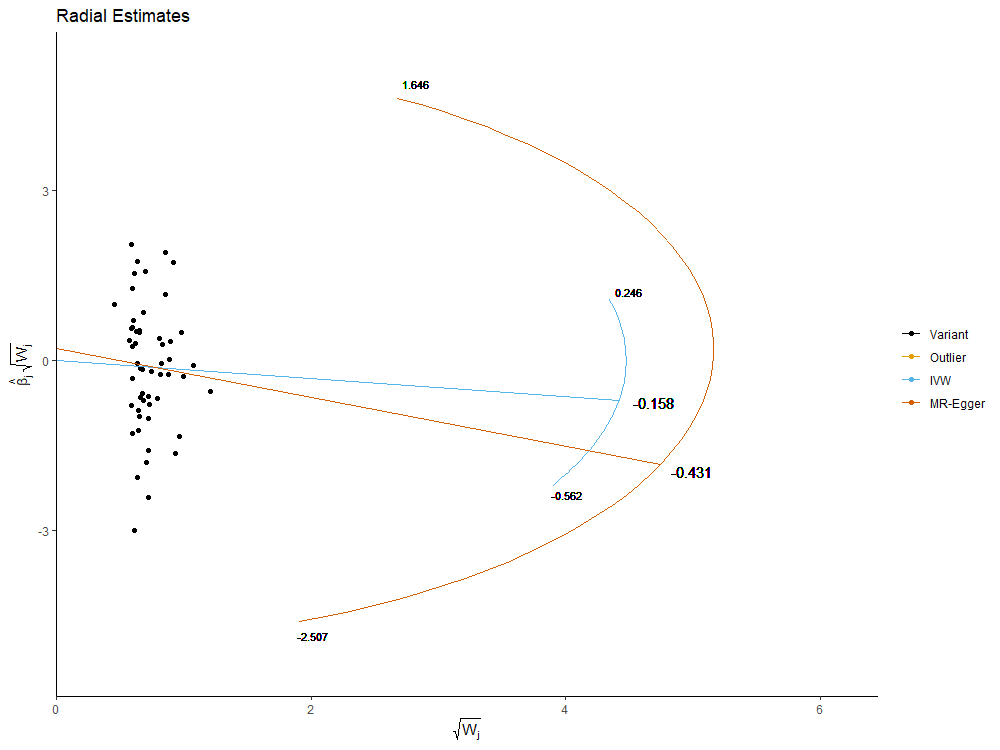

Supplement: S9 Fig — No significant outliers were detected. (TIF) [file pone.0269191.s009.tif]

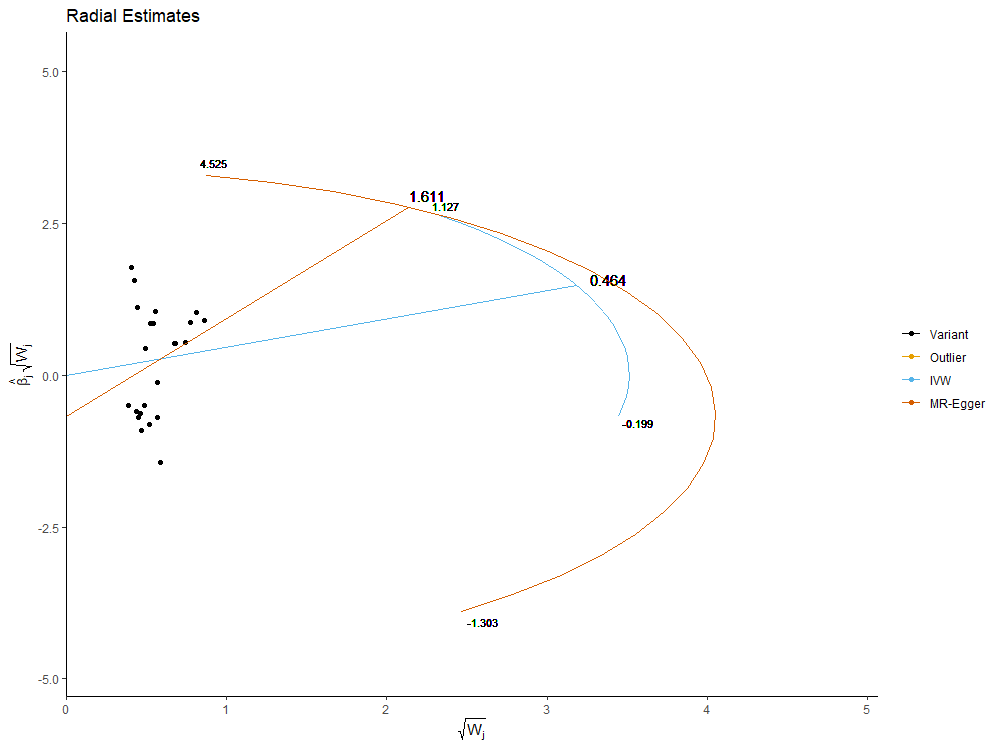

Supplement: S10 Fig — No significant outliers were detected. (TIF) [file pone.0269191.s010.tif]
