# Supplementary material for: Causality of anthropometric markers associated with polycystic ovarian syndrome: Findings of a Mendelian randomization study
Source: PLoS One. 2022 Jun 9;17(6):e0269191. doi: 10.1371/journal.pone.0269191 (PMC9182303; doi:10.1371/journal.pone.0269191)
Supplement: S5 Table — (DOCX) [file pone.0269191.s015.docx]

| **Exposure** | **MR analysis** | **Causal estimate** | **SD** | **T-statistic** | ***p*-value** | **Global test RSS** | **Global test *p*-value** |
| --- | --- | --- | --- | --- | --- | --- | --- |
| Weight | Raw | 0.9890955 | 0.4847096 | 2.040594 | 0.06857919 | 18.96338 | 0.144 |
|  | Outlier-corrected | NA | NA | NA | NA |  |  |
| Height | Raw | -0.1580641 | 0.2012839 | -0.7852793 | 0.4357863 | 66.63444 | 0.14 |
|  | Outlier-corrected | NA | NA | NA | NA |  |  |
| BMI | Raw | **1.40239** | 0.2851014 | 4.918917 | **1.924365E-05** | 36.54108 | 0.584 |
|  | Outlier-corrected | NA | NA | NA | NA |  |  |
| WC | Raw | **1.459643** | 0.4681202 | 3.118095 | **0.006257063** | 28.00698 | 0.136 |
|  | Outlier-corrected | NA | NA | NA | NA |  |  |
| HC | Raw | **1.459643** | 0.4681202 | 3.118095 | **0.006257063** | 28.00698 | 0.116 |
|  | Outlier-corrected | NA | NA | NA | NA |  |  |
| WHR | Raw | 0.4641788 | 0.3197633 | 1.451632 | 0.1607161 | 18.41512 | 0.774 |
|  | Outlier-corrected | NA | NA | NA | NA |  |  |

**S5 Table: Summary of results from Mendelian Randomization Pleiotropy RESidual Sum and Outlier (MRPRESSO) analyses for assessing horizontal pleiotropy and outliers**

**BMI = body mass index; HC = hip circumference; RSS = residual sum of squares SD = standard deviation; WC = waist circumference; WHR = waist-to-hip ratio**
