# Supplementary material for: Causality of anthropometric markers associated with polycystic ovarian syndrome: Findings of a Mendelian randomization study
Source: PLoS One. 2022 Jun 9;17(6):e0269191. doi: 10.1371/journal.pone.0269191 (PMC9182303; doi:10.1371/journal.pone.0269191)
Supplement: S6 Table — (DOCX) [file pone.0269191.s016.docx]

**S6 Table: Congruent evidence from other studies supporting for putative mechanistic roles of the shared and non-shared genes underlying the causal associations between anthropometric markers of central- and/or general obesity and PCOS.**

| **Gene** | **Evidence on putative roles in PCOS pathogenesis from contemporary studies** |
| --- | --- |
| ADCY3 | An experimental mouse model study found that in rats with dihydrotestosterone (DHT)-induced polycystic ovary syndrome (PCOS), manual acupuncture-based stimulation led to faster glucose clearance and modified mainly gene expression in mesenteric adipose tissue including increased ADCY3 (Johansson et al., 2013). |
| **BCDIN3D*** | **BCDIN3D gene was found to be crucial for female fertility and miRNA and mRNA profiles in Drosophila ovaries. This study revealed BCDIN3D's biological role in female fertility and its molecular role in defining miRNA and mRNA profiles in ovaries (Zhu et al., 2019).** |
| **BDNF*** | **A study revealed that BDNF levels in plasma and in follicular fluid were higher in women with PCOS than in healthy controls, indicating that high levels of luteinizing hormone, probably increase the secretion of BDNF in PCOS (Russo et al., 2012). Another in-vitro study revealed effects of BDNF on oocyte maturation and embryonic development in a rat model of PCOS (Zhang et al., 2016).** |
| **DIO2*** | **An experimental study found that DIO2 disruption in zebrafish disturbs both male and female reproduction, linking the loss of DIO2 activity and the resulting hypothyroidism to reproductive dysfunction (Houbrechts et al., 2019).** |
| ETV5 | A mouse model study reported that complex ovarian defects lead to infertility in ETV5−/− female mice suggesting that ETV5 is important for the developmental competence of germ cells and the regulation of responses to steroid hormones in female mice (Eo et al., 2011). |
| **FANCL*** | **A study revealed that FANCL mutations are potentially causative for premature ovarian insufficiency by disrupting DNA damage repair processes (Yang et al., 2020).** |
| FTO | A study found that rs1421085, rs17817449, and rs8050136 variants of the FTO gene were associated with PCOS susceptibility and hyperandrogenemia in young Korean women, which may be mediated through an effect of BMI (Song et al., 2014). Another study revealed that FTO gene variant rs9939609 is associated with hyperandrogenemia and metabolic manifestations of PCOS among women of Sri Lankan descent with the well-characterized phenotype (Branavan et al., 2020). Another study found that FTO gene variation might influence the baseline lipid oxidation in PCOS patients, which might potentially be one of the mechanisms explaining the impact of the FTO gene on body weight in PCOS (Kowalska et al., 2012). |
| **GNPDA2*** | **An experimental study revealed that GNPDA2 may be a critical gene for lipid and glucose metabolism, and the expression level of GNPDA2 alters the transcriptome profile of human adipose-derived mesenchymal stem cells leading to insulin resistance – a cardinal feature of PCOS (Wu et al., 2019). A mouse model study revealed that CNS GNPDA2 does not control appetite, but regulates glucose homeostasis (Gutierrez-Aguilar et al., 2021), which therefore may have mechanistic roles in PCOS pathogenesis, via insulin signaling and glucose regulation pathways.** |
| GPR61 | A mouse model study found that GPR61-deficient mice had obesity associated with hyperphagia, suggesting that GPR61 is involved in the regulation of food intake and body weight, and may have mechanistic roles in PCOS associated with these pathways (Nambu et al., 2011). An integrated epigenomic analysis for T2D susceptibility loci in monozygotic twins found signals specific to T2D-discordant twins in the GPR61, suggesting putative pathobiological roles of GPR61 gene leading to hyperglycemia/insulin resistance – an integral feature of PCOS (Yuan et al., 2014) |
| HMGA2 | An invitro experiment found that the HMGA2/IMP2 pathway was activated in women with PCOS and promoted the proliferation of granulosa cells, providing new insights into the dysfunction of granulosa cells in PCOS (Li et al., 2019). |
| LMX1B | A study revealed that LMX1B rs10733682 polymorphism interacts with macronutrients and dietary Patterns on the risk of obesity (Zhu et al., 2020). This may be a putative pathway through which LMX1B contributes to PCOS via inducing the risk of obesity. |
| **MC4R*** | **A mouse model study found that MC4R downregulation causes abnormal reproductive neuroendocrine profile in female mice. This study indicated that MC4R plays an important role in regulating luteinizing hormone (LH) release, ovulation and reproductive ability probably via hyperphagia-induced obesity (Chen et al., 2017).** |
| MTCH2 | A study concluded that MTCH2 is an obesity susceptibility gene, which induces alteration of fatty acid metabolism in transgenic mice (Bar-Lev et al., 2016), and its role in PCOS might be via augmenting obesity risk. Another mouse model study revealed that the loss of muscle MTCH2 increases whole-body energy utilization and protects from diet-induced obesity (Buzaglo-Azriel et al., 2017) – again indicating pro-obesity role of this gene which may be the pathway through which it contributes to PCOS pathogenesis. |
| **NEGR1*** | **A study found that the obesity‐associated gene NEGR1 regulates aspects of energy balance in rat hypothalamic areas (Boender et al., 2014) while another study revealed that the obesity gene NEGR1 is associated with white matter integrity in healthy young adults (Dennis et al., 2014). Therefore, this gene may partake in ensuing obesity – a cardinal sign of PCOS.** |
| NPC1 | A study found that NPC1 is highly expressed in human white adipose tissue adipocytes with increased levels in obese, suggesting that NPC1 may play a role in adipocyte processes underlying obesity (Bambace et al., 2013). Another study revealed that rare loss-of-function variants in NPC1 predispose to human obesity (Liu et al., 2017). A study concluded that NPC gene interacts with a high-fat diet to promote weight gain through differential regulation of central energy metabolism pathways (Castillo et al., 2017). Together, such obesity-inducing mechanisms driven by NPC1 upregulation might be at play in the onset of PCOS. |
| PDK4 | PDK4 is a key gene regulating insulin signaling and involved in the onset of insulin resistance – a cardinal sign of PCOS. For example, a study found that genetic inactivation of PDK4 mediated pyruvate dehydrogenase kinases improves hepatic insulin resistance (Tao et al., 2013) while another study revealed that insulin regulation of skeletal muscle PDK4 mRNA expression is impaired in acute insulin-resistant states (Kim et al., 2006). Therefore, PDK4 may be involved in ensuing insulin resistance observed in women with PCOS. |
| RPGRIP1L | A study found that RPGRIP1L, a component of the primary cilium, has key roles in adipocyte development and function (Carli et al., 2018), while primary cilia themselves are considered critical regulators of white adipose tissue expansion (Hilgendorf, 2021). Adiposity is frequently observed in PCOS. This gene therefore may have a mechanistic role promoting adiposity in the development of PCOS. |
| **SEC16B*** | **SEC16B gene has putative mechanistic roles in obesity, as evidenced by multiple studies (Lv et al., 2015; Sahibdeen et al., 2018; Fu et al., 2016) and likely imparts its effects on inducing obesity in PCOS.** |
| **SH2B1*** | **SH2B1 has contributory roles in both obesity and insulin resistance, as demonstrated by multiple studies (Jiang et al., 2020; Desbuquois et al., 2013; Rui, 2014; Ren et al., 2007). Therefore, SH2B1 may be involved in the development of insulin resistance and obesity observed among women with PCOS.** |
| **TFAP2B*** | **There is evidence supporting for putative roles of TFAP2B for adiposity, obesity and insulin resistance (Nordquist et al., 2009; Joost et al., 2019) – key features of PCOS.** |
| TLR4 | A study found that TLR4-associated IRF-7 and NFκB signaling act as a molecular link between androgen and metformin activities and cytokine synthesis in the PCOS endometrium (Hu et al., 2021). Another study found that TLR4 inhibits estradiol secretion via NF-κB signaling in human granulosa cells. Findings of this study suggest that functions regulated by TLR4 has important implications for the regulation of ovarian pathophysiology (Guan et al., 2021). Another study discovered that mir-let-7d-3p inhibits granulosa cell proliferation by targeting TLR4 in PCOS (Wu et al., 2021). |
| **TMEM18*** | **As shown by multiple studies TMEM has roles regulating adipogenesis, obesity, appetite, and body weight (Larder et al., 2017; Landgraf et al., 2020). This gene’s role in PCOS might therefore involve these pathways.** |
| ZZZ3 | **A genome-wide association study revealed ZZZ3 is associated with obesity outcomes (Berndt et al., 2013). Therefore, this gene might contribute to the development of obesity that is observed in PCOS.** |

***indicates genes shared by anthropometric markers of both central- and general obesity that were significantly causally associated with PCOS as per univariable 2SMR analyses.**

**References**

Bambace C, Dahlman I, Arner P, Kulyté A. NPC1 in human white adipose tissue and obesity. BMC Endocr Disord. 2013;13:5. <https://doi.org/10.1186/1472-6823-13-5>

Bar-Lev Y, Moshitch-Moshkovitz S, Tsarfaty G, Kaufman D, Horev J, Resau JH, et al. Mimp/Mtch2, an Obesity Susceptibility Gene, Induces Alteration of Fatty Acid Metabolism in Transgenic Mice. PLoS One. 2016;11(6):e0157850. <https://doi.org/10.1371/journal.pone.0157850>

Berndt SI, Gustafsson S, Mägi R, Ganna A, Wheeler E, Feitosa MF, et al. Genome-wide meta-analysis identifies 11 new loci for anthropometric traits and provides insights into genetic architecture. Nat Genet. 2013;45(5):501-12. <https://doi.org/10.1038/ng.2606>

Boender AJ, van Gestel MA, Garner KM, Luijendijk MC, Adan RA. The obesity-associated gene Negr1 regulates aspects of energy balance in rat hypothalamic areas. Physiol Rep. 2014;2(7):e12083.

Branavan U, Wijesundera S, Chandrasekaran V, Arambepola C, Wijeyaratne C. In depth analysis of the association of FTO SNP (rs9939609) with the expression of classical phenotype of PCOS: a Sri Lankan study. BMC Med Genet. 2020;21(1):30. <https://doi.org/10.1186/s12881-020-0961-1>

Buzaglo-Azriel L, Kuperman Y, Tsoory M, Zaltsman Y, Shachnai L, Zaidman SL, et al. Loss of Muscle MTCH2 Increases Whole-Body Energy Utilization and Protects from Diet-Induced Obesity. Cell Rep. 2017;18(5):1335-1336.

Carli JFM, LeDuc CA, Zhang Y, Stratigopoulos G, Leibel RL. The role of Rpgrip1l, a component of the primary cilium, in adipocyte development and function. FASEB J. 2018;32(7):3946-3956. <https://doi.org/10.1096/fj.201701216R>

Castillo JJ, Jelinek D, Wei H, Gannon NP, Vaughan RA, Horwood LJ, et al. The Niemann-Pick C1 gene interacts with a high-fat diet to promote weight gain through differential regulation of central energy metabolism pathways. Am J Physiol Endocrinol Metab. 2017;313(2):E183-E194. <https://doi.org/10.1152/ajpendo.00369.2016>

Chen X, Huang L, Tan HY, Li H, Wan Y, Cowley M, et al. Deficient melanocortin-4 receptor causes abnormal reproductive neuroendocrine profile in female mice. Reproduction. 2017 Mar;153(3):267-276. <https://doi.org/10.1530/REP-16-0341>

Dennis EL, Jahanshad N, Braskie MN, Warstadt NM, Hibar DP, Kohannim O, et al. Obesity gene NEGR1 associated with white matter integrity in healthy young adults. Neuroimage. 2014;102 Pt 2(0 2):548-57. <https://doi.org/10.1016/j.neuroimage.2014.07.041>

Desbuquois B, Carré N, Burnol AF. Regulation of insulin and type 1 insulin-like growth factor signaling and action by the Grb10/14 and SH2B1/B2 adaptor proteins. FEBS J. 2013;280(3):794-816. <https://doi.org/10.1111/febs.12080>

Eo J, Shin H, Kwon S, Song H, Murphy KM, Lim JH. Complex ovarian defects lead to infertility in Etv5-/- female mice. Mol Hum Reprod. 2011 Sep;17(9):568-76. <https://doi.org/10.1093/molehr/gar021>

Fu LW, Zhang MX, Gao LW, Mi J. [Association between SEC16B polymorphisms and body mass index variation or risk of obesity: a Meta-analysis]. Zhonghua Liu Xing Bing Xue Za Zhi. 2016;37(9):1288-1295. DOI: 10.3760/cma.j.issn.0254-6450.2016.09.021

Guan HY, Xia HX, Chen XY, Wang L, Tang ZJ, Zhang W. Toll-Like Receptor 4 Inhibits Estradiol Secretion via NF-κB Signaling in Human Granulosa Cells. Front Endocrinol (Lausanne). 2021;12:629554.

Gutierrez-Aguilar R, Grayson BE, Kim DH, Yalamanchili S, Calcagno ML, Woods SC, et al. CNS GNPDA2 Does Not Control Appetite, but Regulates Glucose Homeostasis. Front Nutr. 2021;8:787470. <https://doi.org/10.3389/fnut.2021.787470>

Hilgendorf KI. Primary Cilia Are Critical Regulators of White Adipose Tissue Expansion. Front Physiol. 2021;12:769367. <https://doi.org/10.3389/fphys.2021.769367>

Houbrechts AM, Van Houcke J, Darras VM. Disruption of deiodinase type 2 in zebrafish disturbs male and female reproduction. J Endocrinol. 2019:JOE-18-0549.R3. <https://doi.org/10.1530/JOE-18-0549>

Hu M, Zhang Y, Li X, Cui P, Sferruzzi-Perri AN, Brännström M, etal. TLR4-Associated IRF-7 and NFκB Signaling Act as a Molecular Link Between Androgen and Metformin Activities and Cytokine Synthesis in the PCOS Endometrium. J Clin Endocrinol Metab. 2021;106(4):1022-1040. <https://doi.org/10.1210/clinem/dgaa951>

Jiang L, Su H, Wu X, Shen H, Kim MH, Li Y, et al. Leptin receptor-expressing neuron Sh2b1 supports sympathetic nervous system and protects against obesity and metabolic disease. Nat Commun. 2020;11(1):1517. <https://doi.org/10.1038/s41467-020-15328-3>

Johansson J, Mannerås-Holm L, Shao R, Olsson A, Lönn M, Billig H, et al. Electrical vs manual acupuncture stimulation in a rat model of polycystic ovary syndrome: different effects on muscle and fat tissue insulin signaling. PLoS One. 2013;8(1):e54357. <https://doi.org/10.1371/journal.pone.0054357>

Joost U, Villa I, Comasco E, Oreland L, Veidebaum T, Harro J. Association between Transcription Factor AP-2B genotype, obesity, insulin resistance and dietary intake in a longitudinal birth cohort study. Int J Obes (Lond). 2019;43(10):2095-2106. <https://doi.org/10.1038/s41366-019-0396-y>

Kim YI, Lee FN, Choi WS, Lee S, Youn JH. Insulin regulation of skeletal muscle PDK4 mRNA expression is impaired in acute insulin-resistant states. Diabetes. 2006;55(8):2311-7. <https://doi.org/10.2337/db05-1606>

Kowalska I, Adamska A, Malecki MT, Karczewska-Kupczewska M, Nikolajuk A, Szopa M, et al. Impact of the FTO gene variation on fat oxidation and its potential influence on body weight in women with polycystic ovary syndrome. Clin Endocrinol (Oxf). 2012;77(1):120-5. <https://doi.org/10.1111/j.1365-2265.2012.04379.x>

Landgraf K, Klöting N, Gericke M, Maixner N, Guiu-Jurado E, Scholz M, et al. The Obesity-Susceptibility Gene TMEM18 Promotes Adipogenesis through Activation of PPARG. Cell Rep. 2020;33(3):108295. <https://doi.org/10.1016/j.celrep.2020.108295>

Larder R, Sim MFM, Gulati P, Antrobus R, Tung YCL, Rimmington D, et al. Obesity-associated gene TMEM18 has a role in the central control of appetite and body weight regulation. Proc Natl Acad Sci U S A. 2017;114(35):9421-9426. <https://doi.org/10.1073/pnas.1707310114>

Li M, Zhao H, Zhao SG, Wei DM, Zhao YR, Huang T, et al. The HMGA2-IMP2 Pathway Promotes Granulosa Cell Proliferation in Polycystic Ovary Syndrome. J Clin Endocrinol Metab. 2019;104(4):1049-1059. <https://doi.org/10.1210/jc.2018-00544>

Liu R, Zou Y, Hong J, Cao M, Cui B, Zhang H, et al. Rare Loss-of-Function Variants in NPC1 Predispose to Human Obesity. Diabetes. 2017;66(4):935-947. <https://doi.org/10.2337/db16-0877>

Lv D, Zhang DD, Wang H, Zhang Y, Liang L, Fu JF, et al. Genetic variations in SEC16B, MC4R, MAP2K5 and KCTD15 were associated with childhood obesity and interacted with dietary behaviors in Chinese school-age population. Gene. 2015;560(2):149-55. <https://doi.org/10.1016/j.gene.2015.01.054>

Nambu H, Fukushima M, Hikichi H, Inoue T, Nagano N, Tahara Y, et al. Characterization of metabolic phenotypes of mice lacking GPR61, an orphan G-protein coupled receptor. Life Sci. 2011;89(21-22):765-72. <https://doi.org/10.1016/j.lfs.2011.09.002>

Nordquist N, Göktürk C, Comasco E, Eensoo D, Merenäkk L, Veidebaum T, et al. The transcription factor TFAP2B is associated with insulin resistance and adiposity in healthy adolescents. Obesity (Silver Spring). 2009 Sep;17(9):1762-7. <https://doi.org/10.1038/oby.2009.83>

Ren D, Zhou Y, Morris D, Li M, Li Z, Rui L. Neuronal SH2B1 is essential for controlling energy and glucose homeostasis. J Clin Invest. 2007 Feb;117(2):397-406. <https://doi.org/10.1172/JCI29417>

Rui L. SH2B1 regulation of energy balance, body weight, and glucose metabolism. World J Diabetes. 2014;5(4):511-26. DOI: 10.4239/wjd.v5.i4.511

Russo N, Russo M, Daino D, Bucci F, Pluchino N, Casarosa E, et al. Polycystic ovary syndrome: brain-derived neurotrophic factor (BDNF) plasma and follicular fluid levels. Gynecol Endocrinol. 2012 Apr;28(4):241-4. doi: 10.3109/09513590.2011.613969. PMID: 22420627. <https://doi.org/10.3109/09513590.2011.613969>

Sahibdeen V, Crowther NJ, Soodyall H, Hendry LM, Munthali RJ, Hazelhurst S, et al. Genetic variants in SEC16B are associated with body composition in black South Africans. Nutr Diabetes. 2018;8(1):43. <https://doi.org/10.1038/s41387-018-0050-0>

Song DK, Lee H, Oh JY, Hong YS, Sung YA. FTO Gene Variants Are Associated with PCOS Susceptibility and Hyperandrogenemia in Young Korean Women. Diabetes Metab J. 2014;38(4):302-10. <https://doi.org/10.4093/dmj.2014.38.4.302>

Tao R, Xiong X, Harris RA, White MF, Dong XC. Genetic inactivation of pyruvate dehydrogenase kinases improves hepatic insulin resistance induced diabetes. PLoS One. 2013;8(8):e71997. <https://doi.org/10.1371/journal.pone.0071997>

Wu L, Ma F, Zhao X, Zhang MX, Wu J, Mi J. GNPDA2 Gene Affects Adipogenesis and Alters the Transcriptome Profile of Human Adipose-Derived Mesenchymal Stem Cells. Int J Endocrinol. 2019;2019:9145452. <https://doi.org/10.1155/2019/9145452>

Wu W, Duan C, Lv H, Song J, Cai W, Fu K, et al. MiR-let-7d-3p inhibits granulosa cell proliferation by targeting TLR4 in polycystic ovary syndrome. Reprod Toxicol. 2021;106:61-68. <https://doi.org/10.1016/j.reprotox.2021.10.003>

Yang Y, Guo T, Liu R, Ke H, Xu W, Zhao S, et al. FANCL gene mutations in premature ovarian insufficiency. Hum Mutat. 2020;41(5):1033-1041. <https://doi.org/10.1002/humu.23997>

Yuan W, Xia Y, Bell CG, Yet I, Ferreira T, Ward KJ, et al. An integrated epigenomic analysis for type 2 diabetes susceptibility loci in monozygotic twins. Nat Commun. 2014;5:5719. <https://doi.org/10.1038/ncomms6719>

Zhang Q, Liu D, Zhang M, Li N, Lu S, Du Y, et al. Effects of brain-derived neurotrophic factor on oocyte maturation and embryonic development in a rat model of polycystic ovary syndrome. Reprod Fertil Dev. 2016 Oct;28(12):1904-1915. <https://doi.org/10.1071/RD15131>

Zhu L, Liao SE, Ai Y, Fukunaga R. RNA methyltransferase BCDIN3D is crucial for female fertility and miRNA and mRNA profiles in Drosophila ovaries. PLoS One. 2019;14(5):e0217603. <https://doi.org/10.1371/journal.pone.0217603>

Zhu Q, Xue K, Guo HW, Yang YH. LMX1B rs10733682 Polymorphism Interacts with Macronutrients, Dietary Patterns on the Risk of Obesity in Han Chinese Girls. Nutrients. 2020;12(5):1227. <https://doi.org/10.3390/nu12051227>
